# A scoping review of continuing education models and statutory requirements for pharmacists globally

**DOI:** 10.1186/s12909-024-05322-4

**Published:** 2024-03-27

**Authors:** Sholene Ballaram, Velisha Perumal-Pillay, Fatima Suleman

**Affiliations:** 1 Discipline of Pharmaceutical Science, College of Health Science, University of KwaZulu-Natal, Private Bag X54001, 4000 Durban, South Africa; 2 School of Health Sciences, College of Health Science, University of KwaZulu-Natal, Private Bag X54001, 4000 Durban, South Africa

**Keywords:** Pharmacists, Continuing professional education (CPE), Continuing professional development (CPD), Lifelong learning (LLL), Models

## Abstract

**Background:**

In the dynamic field of pharmacy amongst a diverse array of countries with disparate income levels, pharmacists play a pivotal role in integrating emerging scientific knowledge into their practice while adapting to evolving therapeutic interventions and expanding service delivery responsibilities. Lifelong Learning (LLL) is cultivated through continuing professional education (CPE) and continuing professional development (CPD), indispensable components ensuring sustained professional competence and heightened patient care quality. The global landscape witnesses diverse LLL activities tailored to pharmacists’ learning needs and preferences. This scoping review maps and synthesises a comprehensive global perspective on the existing knowledge regarding CPE/CPD models, statutory requirements, and pharmacists’ preferences for LLL activities.

**Objective:**

To comprehensively investigate global models of CPE/CPD for pharmacists’ and examine the statutory requirements governing pharmacists’ registration and licensure.

**Method:**

A literature search of PubMed, Google Scholar, Web of Science, and the University of KwaZulu-Natal library search engine was undertaken for studies between January 2012 and February 2023. The article selection and reporting followed the recommendations made by PRISMA (Preferred Reporting Items of Systematic Reviews and Meta-Analyses) guidelines. The articles were tabulated based on their respective country’s income level, continuing education models employed, country-specific statutory requirements, and pharmacists’ preferences for LLL activities.

**Results:**

Of the initial 3974 publications identified through the database search, 24 studies met the review criteria. The majority of the articles originated from high-income countries (HICs) (14/24, 58.3%), and most employed the mandatory CPD points system (21/24, 87.5%). However, in some HICs and upper-middle income countries (UMICs), the CPE/CPD is non-mandatory. While most countries (19/24, 79.2%) offer various LLL formats, the preference of pharmacists remains primarily face-to-face learning (13/24, 54.2%). However, workplace learning (3/24, 12.5%) and blended learning (7/24, 29.1%) are mentioned in some studies.

**Conclusion:**

Diverse models of CPE/CPD alongside statutory requirements persist globally and evolve, shaped by varied implementation experiences. HICs lead in CPD models, while the implementation in low- and middle-income countries (LMICs) and low-income countries (LICs) requires further exploration for inclusivity and effectiveness. A few UMICs are either initiating or in early stages of implementing the CPD models. Structured planning for LLL activities is increasingly a global requirement for pharmacists’ licensure. The essential progression of pharmacy practice in developing healthcare systems necessitates a mandatory CPD model. Ongoing research is crucial to fortify the implementation, align and unify the CPD model with evolving pharmacy profession needs.

## Introduction

### Background

The pharmacy is often the first point of contact for a patient or a caregiver within the healthcare system [1]. The healthcare environment is experiencing ongoing transformation, medical upgrades and technological advancements. Simultaneously, the evolution of pharmacy practice emphasises that the role of pharmacists in the healthcare system has transformed globally [2]. Thus, continuing education, professional development and training are fundamental in building a competent pharmaceutical workforce to satisfy the public’s healthcare requirements.

Continuing professional education (CPE) and continuing professional development (CPD) are increasingly a requirement in most countries for pharmacists’ continued registration [3]. CPE has been emphasised in pharmacy practice much longer than CPD. CPE refers to a structured learning experience by participating in educational and training sessions, including modules and activities and recording hours of education received [4]. 

In the early twentieth century, the pharmacy profession adopted CPD to ensure professional competence. In 2002, the International Pharmaceutical Federation (FIP) embraced the CPD cycle and outlined it as the responsibility of individual pharmacists for systemic maintenance, development and broadening of knowledge and skills to ensure continuing competence as a professional throughout their careers [5]. 

**Fig. 1:**
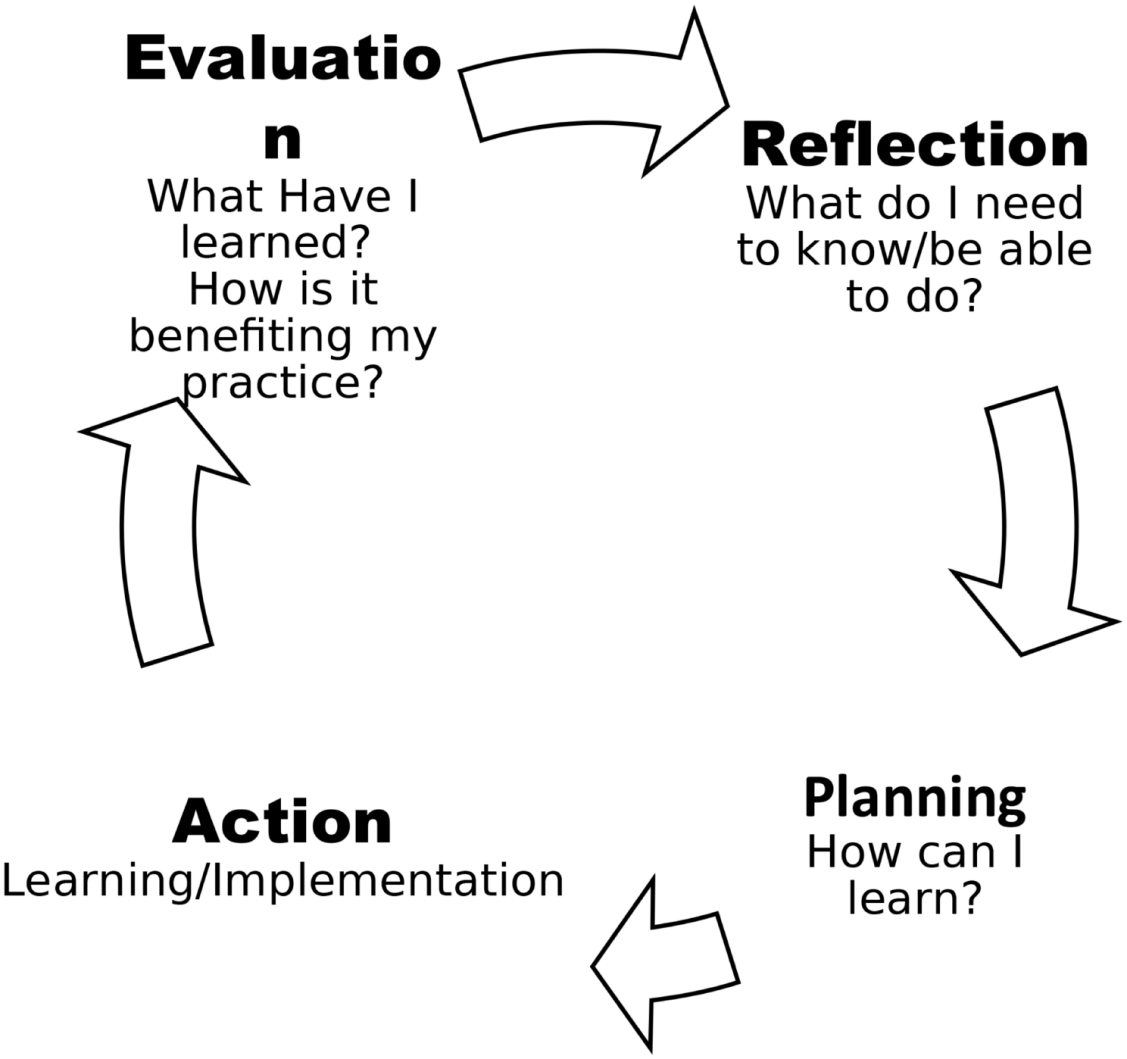
CPD cycle [7]

The CPD cycle, widely adopted by numerous professional entities, finds its roots in Kolb’s (1984) experiential learning theory [6]. This approach intricately weaves theory into practice through iterative cycles of learning, placing reflection at the core of the process. Figure 1 below depicts CPD as a cyclical and self-directed process with four elements that allow pharmacists to [3, 6, 7]:

1. Reflect on their educational needs by identifying what they want to learn and why.
2. Plan the learnings by identifying potential options to achieve their learnings.
3. Action refers to completing the learning for their preferences regarding the activity.
4. Evaluate the impact of these learnings on their professional practice, how they will affect present and future professional practice, and identify future learning needs.

CPD enhances the conventional CPE and allows pharmacists to identify and meet their learning needs to support their professional practice. In addition, CPD provides a greater return on investment than CPE, as CPD focuses on educational context and application and incorporates more elements in its cycle [8, 9]. Austin et al., 2005 suggests that pharmacists may benefit from systematic, structured orientations to CPD and that developing a learning portfolio can address their concerns [8]. Learning portfolios in health professions consists of a series of documents (articles, references), records (certificates, materials of courses attended), logs (descriptions of significant events in practice) and reflections (analysis of the ways in which documents, records and logs have led to new understandings or new ways of practicing) [8]. 

In preparation for a CPD portfolio, the process to document and record the learnings may be time-consuming [10]. CPE and CPD contribute to lifelong learning (LLL) and are essential throughout one’s professional life. LLL is a personal or professional approach to learning that is continuous and self-motivated and can include formal or informal educational interventions [3, 11]. LLL activities may be laborious, inconsistent and occasionally attempted by a healthcare professional to refine their knowledge, skills and professional competence [11]. Hence, CPE and CPD ensure that LLL are accomplished and recorded by accumulating continuing education hours, points or the number of entries required by completing activities related to competency development [12]. In medical education, competency refers to measurable abilities that incorporate essential knowledge and skills that compose the standards and expectations of healthcare professionals, to provide comprehensive and optimal patient care [13]. Hence, the appraisal of the variance in current and desired levels of pharmacist competencies enables the formulation of an individualised learning plan directed towards achieving the desired competency level [13]. 

A global FIP report indicated that CPD has been accepted and fully implemented in most countries while others operated on the formal CPE system [3, 14, 15]. Furthermore, most countries previously used the models centred on the accumulation of hours, points earned and activities related to competency development rather than a structured LLL activity for evidence-based outcomes [16]. In Ghana, LLL practices moved from CPE to the CPD process model [17]. CPD was initially promoted in Canada, the United States, and the United Kingdom [17]. CPD is a vital component in the growth of pharmacy practice to enhance pharmacists’ skill sets through active training and learning. Therefore, its use varies across diverse practice environments [17]. 

A study in the Netherlands, Germany, Great Britain, Canada (Ontario), New Zealand, Australia, France and the United States presented various LLL formats to the pharmacists to deliver CPE and CPD [18], including face-to-face activities such as lectures or workshops and distance learning formats, like printed materials, audio-recording, and the Internet (e-learning, webinars). CPD incorporates a wide range of LLL activities, formal (lectures, interactive sessions, seminars or conferences attendance, distance and online learning offered by accredited bodies) and informal education interventions. Informal educational interventions include audits and feedback, printed materials, academic detailing, outreach visits, reminders and opinion leaders [18]. Globally, there has yet to be a unified view of the core characteristics of LLL for pharmacists.

CPE, CPD and LLL activities are essential for pharmacists to ensure they remain knowledgeable, skilled and capable of providing the best possible care to their patients, to maintain competence, adapt to changes rapidly, ensure patient safety at all times, professional growth, to meet regulatory requirements, enhance patient outcomes, foster professionalism and keep pace with technology in an ever-evolving healthcare landscape.

In low-middle income countries (LMICs) and low-income countries (LICs), a significant gap exists in the training and education of pharmacists. In 2019/2020, South Africa, an upper-middle income country introduced the CPD model to address this issue. However, comprehensive global perspectives on this initiative are lacking in the existing literature, with most studies focusing on high-income countries in their comparative analyses. Few studies have explored LMICs, and a conspicuous absence of reviews evaluating CPD models and LLL activities for pharmacists across income levels is evident. This study aims to fill this gap by scrutinising the CPD models and statutory requirements for pharmacists that is crucial for assessing how regulatory changes align with ongoing advancements in pharmacy practice.

The implications of these differences on pharmacy practice include considerations for resource allocation, patient-centric approaches, and technological integration. Understanding these variations is crucial for refining CPD frameworks globally. The absence of comprehensive reviews, covering a range of countries with differing economic statuses, underscores the necessity of this study. The information generated can serve as a valuable resource for countries looking to refine or establish their CPD models for pharmacists. This research is essential for advancing the understanding of global best practices in pharmacy CPD, fostering international collaboration, and providing actionable insights for improving CPD frameworks.

## Method

### Research design

**Fig. 2:**
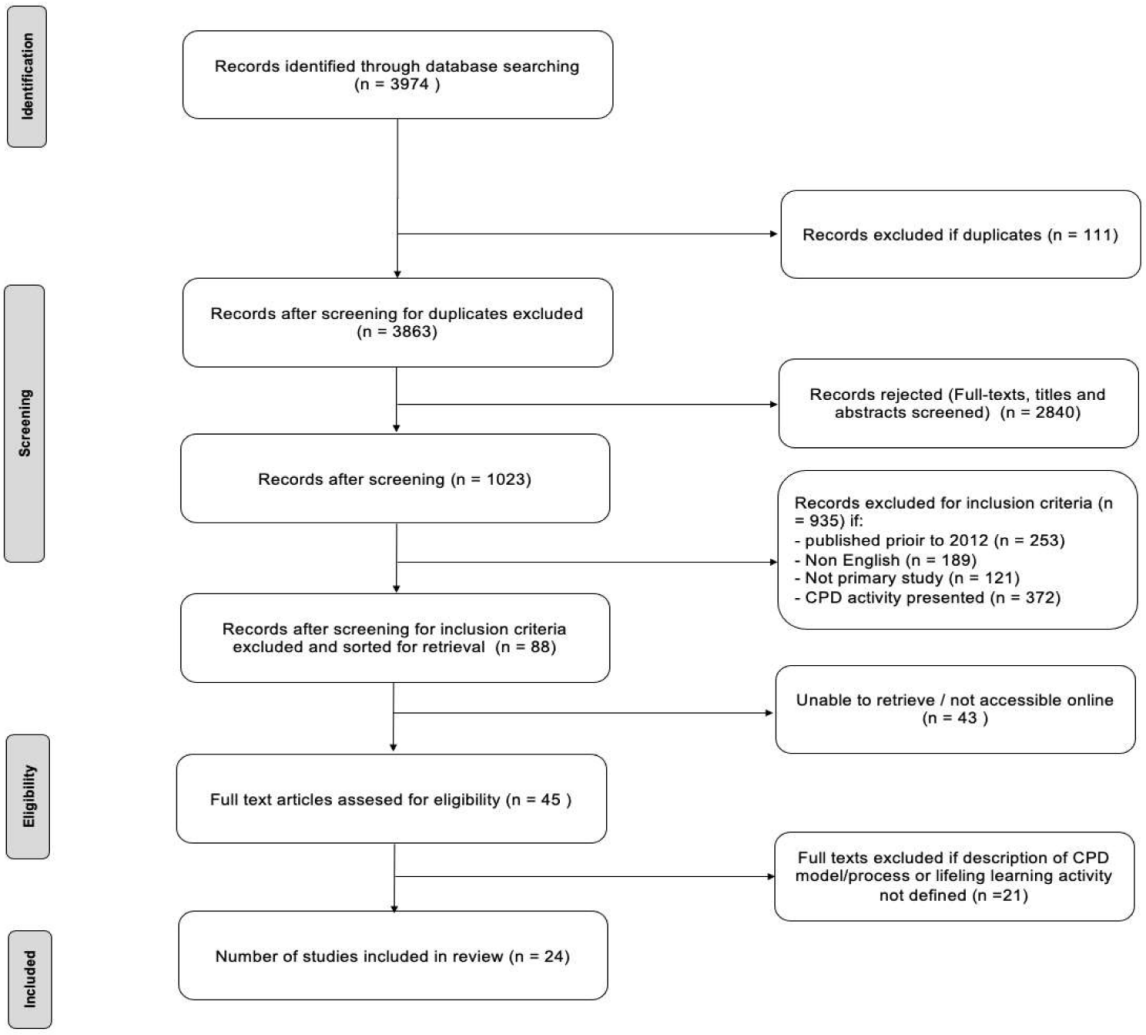
PRISMA flow chart of the search strategy and article selection^3^

The study design adopted was a scoping review approach of the available evidence regarding the current trends in pharmacists’ CPE/CPD models implemented globally. This review was conducted following the six-stage methodology of the Joanna Briggs Institute, based on the original scoping review framework of Arksey and O’Malley [19]. A scoping review is appropriate for this research, whereby a diverse range of literature exists, enabling a comprehensive summary of information to be available from which gaps and focal areas will be identified. The article selection and reporting followed the recommendations made by the Preferred Reporting Items of Systematic Reviews and Meta-Analyses (PRISMA), as depicted in Fig. 2 [19]. 

### Search strategy

A literature search was undertaken in March 2023, to identify papers published between January 2012 and February 2023. The twelve years were chosen based on the significant pharmaceutical and medicinal transformations in experimental continuing education and professional development of pharmacists. The databases searched to obtain papers included PubMed, Google Scholar, Web of Science, and the University of KwaZulu-Natal library search engine.

The researcher developed a search string that combined the following keywords and their synonyms expressed in Boolean values. The search terms included pharmacist/s, continuing professional education (CPE), continuing professional development (CPD), lifelong learning (LLL) and models. The databases and the reference lists of the most relevant studies for this scoping review were searched afterwards. Articles were chosen for this study based on the inclusion and exclusion criteria.

The inclusion criteria for this research encompassed studies conducted globally between January 2012 and February 2023. The study exclusively considered materials presented in the English language only, emphasising a comprehensive analysis of primary studies. Only those studies with easily accessible full-texts were included, specifically targeting research related to Continuing Professional Education (CPE), Continuing Professional Development (CPD) models, and Lifelong Learning (LLL) activities.

Conversely, the exclusion criteria for this research outlined that studies were omitted if they lacked accessibility in full-text versions or were not presented in English. The removal of duplicate articles was a prerequisite for inclusion. Studies were further excluded if they did not qualify as primary studies or lacked descriptions of Continuing Professional Education (CPE), Continuing Professional Development (CPD), or Lifelong Learning (LLL) activities. Additionally, studies involving pharmacy students, other healthcare professionals, or those featuring a presentation of a CPE/CPD activity or similar were not considered for this review. No grey literature was included in this particular review, which may have resulted in some literature being missed, such as those on regulator websites.

### Data collection

The reviewer screened the article titles and abstracts of all identified articles. After applying the inclusion and exclusion criteria, duplicate articles were removed. After initial screening of all extracted articles, full-text articles were reviewed and removed if the primary objectives of the paper were not to investigate models of continuing education and LLL activities for pharmacists. Other researchers validated this screening.

### Data extraction and analysis

Microsoft Excel was used to draw up a data extraction sheet. A table format was used to extract and summarise the data. The extraction sheet depicted in Table 1 provides an analysis of the general information about each article: the author, year of publication, study country, country income level, sample size, method of data collection, country-specific CPE/CPD requirement, statutory requirement, the model used, LLL format, key findings, and contextual factors.

## Results

### Study selection

The key characteristics of the 24 studies included in this scoping review for 2012–2023 are summarised in Table 1. The initial search of the databases yielded 3974 online articles with potentially relevant citations. Following deduplication, primary study selection, full-text, title and abstract screening, 1023 articles met the eligibility criterion. After the second phase of article review for inclusion and exclusion criteria, 88 articles were sorted for further screening. Of these, only 45 full-text articles could be obtained for evaluation. Following data characterisation of the full-text articles, 21 articles were excluded based on the lack of description of the CPE/CPD models, LLL activities or statutory requirements for pharmacists and 24 articles were retained for review. Figure 2 depicts the PRISMA diagram of included and excluded studies to review the CPE/CPD models and LLL activities.

### Study characteristics

The 24 articles that met the inclusion criteria were conducted in sixteen different countries, namely the United Kingdom (UK) (3/24, 12.5%), the United States of America (USA including Trinidad and Tobago) (7/24, 25%), Canada (1/24, 4.2%), Australia (1/24, 4.2%), Europe (Poland/Serbia) (2/24, 8.3%), Africa (Nigeria, Ethiopia, Egypt, Zambia, and Ghana) ( 5/24, 20.8%), Middle East (Kuwait, Saudi Arabia and Lebanon) (4/24, 16.7%) and from different parts of Asia (India, Malaysia, and Pakistan) (3/24, 12.5%) and involved licensed and practising pharmacists only. Table 1 provides a broad overview of the characteristics of each national model with a detailed description of the CPE/CPD, statutory requirements, and LLL activities utilised in the various countries globally.

The 24 articles included collectively reporting on more than ten thousand participants. The number of participants in each study ranged from 12 to a maximum of 3876 in sample size. A quarter of the studies (6/24, 25%) involved less than 100 participants. Most studies (20/24, 83.3%) used the quantitative approach (survey/questionnaire method), whereas a smaller portion (2/24, 8.33%; 2/24, 8.33%) used the qualitative (interviews) and the mixed methods (survey and focus groups/interviews) approach respectively. Most studies (15/24, 62.5%) were published prior to the COVID-19 pandemic.

### Description of CPD/CPE/LLL activities

Only four countries indicated that CPE/CPD practice is non-mandatory for licensed pharmacists in their respective countries (Kuwait, Malaysia, Pakistan and Trinidad and Tobago) [17, 20, 21]. Pharmacists may complete LLL activities voluntarily without submission to the statuary board but pay annual fees for reregistration [17, 20]. CPE was voluntary in some regions of Egypt but mandatory CPD practice in most other parts [22]. Most articles (22/24, 91.7%) discussed CPD as the professional development model.

Some parts of the USA (2/24, 8.3%) practised CPE, while others were transitioning to CPD at the time of the study. The majority (13/24, 54.2%) revealed that pharmacists preferred using face-to-face learning as a form of CPD. In contrast, almost one-third (7/24, 29.1%) preferred a multicomponent approach, blended learning, which often includes e-learning and traditional face-to-face interventions. In the remaining studies (4/24, 16.7%), the pharmacists preferred e-learning. Interestingly, most studies (19/24, 79,2%) used both types of LLL formats (e-learning and face-to-face) at least once in the country, and a small number of studies (3/24, 12.5%) mentioned that pharmacists favoured workplace learning.

**Table 1:** Summary analysis of characteristics of included articles (*n* = 24)

Study Author, Year and Setting	Country	World Bank Classification^a^	Sample size^b^	Data Collection	CPD Requirement	Statutory Requirement^c^	Model Used^d^	Lifelong Learning Format^e^ ^(Most preferred)^	Key Findings	Contextual Factors
Abdullahi, et al.; 2023; [23]	Nigeria.	LMIC	586	Questionnaire	Minimum of 30 credit points per five-year cycle.	M	1	B	Most pharmacists preferred attending seminars, reading journal articles and attending conferences.	The Mandatory CPD (MCPD) Programme has been divided into three modules and points allocated accordingly. The MCPD Curriculum comprises structured courses available at universities and research institutions.
Adhikar, et al.; 2020; [24]	Nepal, India.	LMIC	15	Questionnaire	15 CPD points per five-year cycle.	M	1	B	Pharmacists understand the importance of CPD. Live presentations and handouts are preferred.	There are no structured CPD modules, but the Pharmacy Council of India conducts continuing education programmes nationwide at various approved institutions.
Aldosari et al.; 2020; [20]	Kuwait.	HIC	409	Questionnaire	125 h in five-year cycle.	NM	2	B	Seminars are the most preferred continuing education activity, followed by conferences and then reading journals.	Pharmacists in the governmental sector = no re-licensure or revalidation. Private sector = license renewed every five years. Pharmacists are not required to attend or keep a log of CE or CPD activities, only fee payment.
Alharthi, et al.; 2021; [25]	Saudi Arabia.	HIC	409	Questionnaire	20 h per year.	M	1	C	There should be continuing education opportunities at the workplace.	CPD activities are completed online. Pharmacist’s main reason for attending continuing education events is to obtain a certificate with the hours reflected.
Alkhateeb et al.; 2016; [26]	Texas, USA.	HIC	600	Survey	A total of 30 h per year.	M	1	A	Online continuing education was preferred. Those who liked to attend > 2 h programs were less likely to complete > 50% of the required credit hours through online CE programs.	***** Pharmacists maintain a CPD portfolio online using the worksheets provided to log their learning outcomes and growth.
Aziz et al.; 2013; [21]	Malaysia.	UMIC	3876	Survey	30 CPD points within two years.	NM	1	B	The majority of pharmacists preferred attending workshops and conferences.	CPD activities are recorded online with the title as per listing, category of CPD and points earned. Accreditation and assessment are managed online by assessors.
Buxton et al.; 2012; [27]	Wisconsin, USA.	HIC	50	Survey	A total of 30 h per year.	M	1	A	Most were likely to attend webinars related to their practice field, preferably offered by universities or educational institutions.	***** Pharmacists get approved 1 credit point every 10 h, which does not apply to individuals when the license was first approved.
Chambers et al.; 2013; [28]	Australia.	HIC	68	Survey	40 CPD points annually.	M	1	B	Most pharmacists preferred face-to-face CPD activities, though pharmacists in remote areas felt disadvantaged.	A registering authority in each state and territory in Australia is responsible for registering and regulating pharmacies.
Gelayee, et al.; 2018; [29]	Gondar, Northwest Ethiopia.	LIC	46	Questionnaire	Thirty continuing education units per year.	M	1	B	Interactive workshops were most preferred, whereas internet-based CPD was least preferred.	The Ethiopian Pharmaceutical Association has an online platform, a mobile app with free CPD resources, and free access to journals and articles is hosting free webinars.
Henkel, P.J. and Marvanova, M.; 2018; [30]	USA.	HIC	1239	Survey	A total of 30 h per year.	M	2	C	The most important factor guiding CPE selection is to maintaining licensure, personal interests and self-improvement.	*****
Ibrahim, O., H., M.; 2012; [22]	Egypt.	LMIC	359	Questionnaire	15 CPD Points in a five-year cycle.	M	Some regions have CPE, and others mostly CPD.	B	Pharmacists prefer to attend seminars/workshops and certification programs.	The Egyptian Drug Authority is responsible for continuing pharmacists education and provides relevant activities in partnership with drug companies and the Faculty of Pharmacy.
Iskandar, et al., 2018, [31]	Lebanon.	LMIC	107	Survey	45 credit points in a three-year cycle.	M	1	A	Pharmacists are highly committed to continuing education. Pharmacists preferred online rather than face-to-face and interactive workshops.	One-third of the credits must be allocated to the attendance of seminars.
Johnson, et al.; 2020; [32]	UK.	HIC	159	Survey	Minimum of 9 entries per year to remain on the GPhC register.	M	1	C	Pharmacists preferred blended learning.	The General Pharmaceutical Council (GPhC) regulates the pharmaceutical sector. The Professional Leadership Body (PLB) assumes the role of a professional body for the pharmacy sector. The use of online interactive tools for self-reflection and assessment.
Ladymon, L. B.; 2017; [33]	Tennessee, USA.	HIC	12	Interviews, Focus groups and journal assignments.	A total of 30 h per two-years.	M	2	B	Pharmacists have positive experiences and learnings from CPE. Live interactive lectures were most preferred, followed by online.	***** 15 h may be completed as a home study. The Tennessee Board of Pharmacy accepts courses from providers accredited by ACPE only.
Micallef, et al.; 2020; [34]	UK.	HIC	338	Mixed methods: Interviews and Questionnaires	Minimum of 9 entries per year to remain on the GPhC register.	M	1	B	e-learning is most utilised in training delivery, but face-to-face learning is preferred.	The General Pharmaceutical Council (GPhC) regulates the pharmaceutical sector. The Professional Leadership Body (PLB) assumes the role of a professional body for the pharmacy sector. The use of online interactive tools for self-reflection and assessment.
Mutati, et al.; 2022; [35]	Zambia.	LIC	111	Survey	Minimum of 45 h per three-year cycle.	M	1	C	Blended learning approaches either face-to-face or online, were preferred, coupled with hands-on interactive skills workshops.	The Pharmaceutical Society of Zambia, in collaboration with the Commonwealth Pharmacists Association, offers pharmacists free CPD programmes specially designed for those working in low-resource settings.
Nesterowicz, et al.; 2016; [36]	Poland, Europe.	HIC	113	Survey	100 CPD points per 4 years.	M	1	A	Younger pharmacists participate more frequently in e-learning. Lack of face-to-face contact limited participation in e-learning.	CPD is not integrated into national standards for quality of care. The compliance is not monitored at the regional level.
O’Loan, L.; 2019; [37]	Northern Ireland, UK.	HIC	419	Questionnaire		M	1	C	Workplace learning activities improved professional practice when compared to unstructured learning only.	The Pharmaceutical Society of North Ireland is both a regulator and professional body for pharmacy.
Sacre et al.; 2019; [38]	Lebanon.	LMIC	628	Mixed Methods: Survey & Focus g Groups.	45 credit points in a three-year cycle.	M	1	B	Pharmacists preferred live courses to online, and a small number was not interested in any continuing education. However, the majority used online courses at least once.	One-third of the credits must be allocated to the attendance of seminars.
Shamim, et al.; 2021; [17]	Pakistan (P), Ghana (G), Trinidad& Tobago (T&T).	P = LMIC G = LMIC T&T = UMIC	12	Interviews	P: 40 CPD points per CPD cycle. G: 15 CPD points per year. T&T: 30 points per year.	P = NM G = M T&T = NM	1	C	Pharmacy Councils/Boards in these countries are responsible for developing the CPD model and LLL activities. In Ghana, attending CPD events to renew registration is mandatory but P and T&T pay fees for license renewal.	Pakistan: No continuing education activities are available to motivate pharmacists, especially in remote areas. Ghana: Pharmacists must attend accredited CPD programs for license renewal. T & T: Pharmacists are encouraged to attend courses specific to their license.
Shearer, et al.; 2018; [39]	Canada.	HIC	162	Survey	A minimum of 25 h must be from accredited learning activities.	M	1	C	Practice-based education would help pharmacists apply skills. Workplace learning is pivotal yet needs to be more emphasised in professional development.	Each provincial regulatory authority manages CPD solely. The National Canadian Council for Continuing Education in Pharmacy (CCCEP) accredits all provincial CPD programmes for pharmacists.
Stojkov, et al.; 2022; [40]	Serbia, Europe.	UMIC	565	Survey	6 CPD entries per year.	M	1	B	Most pharmacists were interested in face-to-face workshops followed by case reports and third lectures.	In 2014, the Pharmaceutical Chamber of Serbia adopted the National Competency Framework for evaluation.
Trewet, C. B. and Fjortoft, N.; 2013; [41]	Chicago, USA.	HIC	105	Survey	30 h for every two years.	M	1	B	Pharmacists used worksheets before face-to-face meetings and reported successful application of learning and achievement of the learning plan.	***** The government, universities and colleges offer approved continuing education courses at a cost.
Young, A. M.; 2012; [42]	Massachusetts, USA.	HIC	609	Survey	20 h per year.	M	1	B	Live, face-to-face programs were the preferred format for continuing education.	***** 15 h may be completed as a home study. Online registration and license renewal.

Key:

^a^Country Income Level: HIC = High-income; UMIC = Upper-to-middle-income; LMIC = Low-to-middle-income; LIC = Low-income

^b^Sample size = Number of Pharmacists

^c^Statutory Requirements: M = Mandatory; NM = Non-mandatory

^d^Model Used: 1 = CPD; 2 = CPE

^e^Lifelong Learning Format: A = Online/e-learning; B = Face-to-face; C = Both (e-learning and face-to-face)

^f^USA: Some states are still using the traditional CPE model of development. The Council of Credentialing in Pharmacy (CCP) maintains consistency among the states in pharmacy programmes. The Accreditation Council for Pharmacy Education (ACPE) sets standards and accredits CPD providers and not CPD activities

Table 2 summarises the publication year, country of origin and the other characteristics of the 24 articles. The years of publication ranged from 2012 to 2023, with two-thirds (16/24, 66.7%) of the articles in the latter part of this period (2017–2023). The majority (7/24, 29.2%) of the studies originated from the USA, a high-income country (HIC), followed by Africa (5/24, 20.8%). Other HICs included the UK, Saudi Arabia, Kuwait, Europe (Poland), Canada and Australia. More than half of the articles (14/24, 58.3%) featured HIC. Two articles (2/21, 9.5%) featured low-income countries (LICs), namely Pakistan and Ethiopia. A few articles (3/24, 12.5%) presented data on upper-middle-income countries (UMIC), namely Serbia and Malaysia. Many articles (7/24, 29.2%) included low-middle income countries (LMICs), Nigeria, India, Egypt, Lebanon, Pakistan and Ghana.

**Table 2:** Analysis summary of included studies for CPE/CPD features

Characteristics of included articles	N (%)
**Year of publication**	**24 (100)**
2012–2016	8 (33.3)
2017–2023	16 (66.7)
*COVID-19 pandemic years (2020–2023).	9 (37.5)
**Country classifications by income groups	**26 (100)**
Low income	2 (7.7)
Lower-middle income	7 (26.9)
Upper-middle income	3 (11.5)
High income	14 (53.8)
**Research Method**	**24 (100)**
Quantitative	20 (83.4)
Qualitative	2 (8.3)
Mixed methods	2 (8.3)
**Country statutory requirements	**26 (100)**
Mandatory	22 (84.6)
Non-mandatory/Voluntary	4 (15.4)
**Continuing education model used**	**24 (100)**
Continuing professional education (CPE)	3 (12.5)
Continuing professional development (CPD)	21 (87.5)
**Lifelong learning format /CPD Modality**	**24 (100)**
E-learning only	4 (16.7)
Face-to-face only	13 (54.2)
Blended (Both e-learning and face-to-face)	7 (29.1)

*Covid-19 pandemic period included. **Note: Some articles involve multiple countries in the study

Table 3 outlines the current trends of CPD/CPE between HICs and LMICs, the effective date for implementation and CPE/CPD statutory requirements for pharmacists [14]. CPD has been accepted and fully implemented in most countries, while other LMICs operate on the formal CPE model [3]. A handful of regions among the specific countries are either shifting or considering the shift from CPE to the CPD model. Furthermore, most countries used models centring on the accumulation of points earned (10/24, 41.7%) rather than hours (8/24, 33.3%) and entries (6/24, 25%).

Table 3 shows that licensed pharmacists in Pakistan, Trinidad and Tobago, Kuwait and Malaysia practice non-mandatory CPE/CPD in their respective countries for reregistration [17, 20, 21]. In contrast, Ghana pharmacists need to attend LLL activities to renew their registration as a licensed pharmacist [17]. However, Ghana has no CPD model [17]. Egypt practices CPE voluntarily in some regions and mandatory CPD in others [22]. Despite the country’s income level, all countries have demonstrated an increased focus on ensuring the implementation of processes to strengthen pharmacists continuing education and professional development.

**Table 3:** Current trends of continuing education for pharmacists in HIC and LMICs

Country	CPE/CPD Statutory requirement and effective date
**Current trends of continuing education for pharmacists in HIC**	
United States of America (USA) [15, 26, 27, 30, 33, 41, 42].	CPE has been mandatory since 1965, and most provinces have shifted to CPD since 2002.
Australia [15, 28].	CPD has been mandatory since 2010.
Canada [15, 39]	CPD has been mandatory in some provinces since 2005.
United Arab Emirates, Kuwait, and Saudi Arabia [15, 20, 25]	CPD mandatory, UAE = 2014, Saudi Arabia = 2016 and Kuwait = non-mandatory 2020.
United Kingdom (UK) [32, 37]	CPD mandatory since 2005.
Northern Ireland [37]	CPD mandatory from 01 June 2021.
**Current trends of continuing education for pharmacists in LMICs**	
Nigeria [23]	CPD program launched in 2008.
India [24]	CE was introduced in 1948. Mandatory CPD since 2016.
Egypt [15, 22]	CPD has been mandatory since 2017, while some regions still have voluntary CPE.
Lebanon [31, 38]	Mandatory CPD since 2014.
Ghana [43]	Mandatory since 2013, but no CPD model.
Pakistan, Malaysia, & Trinidad and Tobago [43]	Non-mandatory CPD since 2010.

Most articles (22/24, 92%) highlighted the types of CPD interventions incorporating formal and informal approaches and considered the pharmacists’ preferences for LLL activities. Three key themes have emerged as the focus of these studies:

- **Theme 1**: The awareness and understanding of statutory requirements for the country-specific CPE/CPD model (22/24, 92%) [21–34, 36, 38–42].
- **Theme 2**: The perceived benefits and importance of the continuing education model (18/24, 75%) [17, 20–27, 29–31, 33, 35, 39–42].
- **Theme 3**: Pharmacists’ preferences for LLL activities (22/24, 92%) [17, 20–29, 31, 33–35, 40–42].

Most articles (22/24, 92%) highlighted that many pharmacists in the respective country are informed of the CPE/CPD model. The majority (22/24, 92%) understand the concepts related to continuing education for their professional development and know their country-specific statutory requirements for CPE/CPD. The models used by pharmacists globally varied drastically, with most countries moving towards the CPD model. However, a few regions (Tennessee (USA), Pakistan and Trinidad and Tobago) and other areas in some regions (Egypt) are slowly transitioning between CPE and CPD models. Many studies (18/24, 75%) emphasised the importance and perceived benefit of the CPE/CPD model for self-improvement and professional development.

## Discussion

This review analyses 24 articles on global continuing education and professional development for pharmacists, encompassing diverse countries with varying income levels. The study reveals notable variations in CPE/CPD practices, highlighting disparities in healthcare systems, educational structures, and regulatory frameworks across nations. Despite a literature gap in the comprehensive analysis of pharmacy continuing education, the review sheds light on statutory requirements, diverse LLL interventions and professional development preferences. Economic factors impact the initiation, methodologies, criteria, and standards of continuing education initiatives, fostering substantial variations. The identified differences in resources, recognition of pharmacists’ roles, and emphasis on continuous learning contribute to the observed global variations in CPE/CPD models and statutory requirements.

The landscape of CPD has undergone significant progress in the aftermath of the COVID-19 pandemic. However, the existing published literature falls short in accurately reflecting the current state of CPD, LLL activities and pharmacists preferences. Despite the wealth of information available on continuing education, only a limited number of articles met the search criteria. This scarcity could be attributed to a potential lack of awareness and financial resources dedicated to CPE/CPD in numerous countries, thereby contributing to the observed variations in the utilised models.

Several countries, including Australia, Canada, Britain, parts of the USA, and New Zealand, have embraced the CPD model, as noted by Rouse [3]. Consistent with prior research, our findings underscore that HICs take the lead in CPD initiatives for pharmacists [3, 16, 17, 44]. Specifically, in countries like the USA and UK, characterised by robust healthcare systems, substantial resources, and a heightened recognition of pharmacists’ evolving roles in patient care, CPD initiatives are well-established. The commitment to high-quality educational experiences is evident through investments in education, infrastructure, and technology, leading to the prevalence of face-to-face and blended learning approaches. Additionally, McConnell and colleagues, in a USA study, illustrated positive changes in behaviour and knowledge patterns resulting from the adoption of the CPD model, replacing the traditional CPE model in that setting [44]. 

In the Sub-Saharan Africa, South Africa (SA) an UMIC, the South African Pharmaceutical Council (SAPC) has initiated an outcome-based learning system and a competency framework in 2019/2020 mandated for pharmacist reregistration, which prioritises the quality of learning outcomes and reflective practice over the accumulation of points or hours, aligns with systems implemented in the USA, UK, Saudi Arabia and Canada [15, 29, 35, 44, 45]. Despite challenges, blended and e-learning methods gained prominence. SAPC’s robust technology infrastructure enables remote pharmacists to fulfil CPD requirements through virtual workshops, supported by tools like a CPD guidance document and online resources. (46) The pilot project from 2010 to 2015 paved the way for the 2019/2020 change, requiring at least six CPD activities annually for SAPC reregistration. This shift and its impact on the CPD cycle warrant exploration and comparison with global models [35]. 

Conversely, the inclusion of countries from Africa, the Middle East, and various parts of Asia, characterised by LMICs and LICs or those with resource constraints, provides a valuable perspective on regions with diverse socio-economic conditions. LLL activities in these regions may encounter challenges related to accessibility, funding, and infrastructure [17, 21–23, 35]. Factors such as limited resources, infrastructure challenges, and varying degrees of government investment in healthcare and education can impact the nature and accessibility of CPD programs [17, 22–24, 29, 31, 35]. The observed preference for workplace learning in some instances may be indicative of resource-conscious approaches to professional development. These countries may struggle to provide adequate funding for CPD programs, hindering access to quality education and limiting the adoption of advanced learning technologies. In regions with limited internet connectivity or outdated technological resources, pharmacists may face difficulties in accessing online or technology-driven CPD initiatives.

The identification of countries where CPE/CPD are non-mandatory for re-registration purposes, such as Kuwait, Malaysia, Pakistan, and Trinidad and Tobago, underscores the flexibility and adaptability of continuing education models [17, 20, 21]. In these contexts, pharmacists may engage in voluntary LLL activities, potentially influenced by the economic considerations and regulatory frameworks of their respective countries. In these countries and many others, due to the expiatory percentages in the country, cultural and linguistic differences may impact the effectiveness of LLL activities. Language barriers can hinder the understanding and implementation of new concepts, while varying cultural norms may influence the acceptance of certain educational approaches.

The impact of these variations in CPD practices is multifaceted [44]. Firstly, it can affect the quality of healthcare services provided by pharmacists. Disparities in CPD opportunities may lead to gaps in knowledge and skills, potentially compromising patient care. Secondly, the variations can impact professional mobility and recognition. Pharmacists trained in one country may find it challenging to adapt to the CPD requirements of another, affecting their ability to practice internationally [45]. Pharmacists may face challenges in navigating diverse regulations, adapting to changes, and ensuring compliance with varying licensing and certification standards. This can have implications for workforce mobility and global collaboration in healthcare.

Geographical factors, such as rural-urban divides, may affect the accessibility of LLL activities. Pharmacists in remote areas may have limited access to educational resources and networking opportunities compared to their urban counterparts. Moreover, the observed variations may contribute to inequalities in the professional development opportunities available to pharmacists globally. This can perpetuate disparities in healthcare outcomes and hinder efforts to achieve a standardised level of pharmaceutical care on an international scale.

The majority of the reviewed articles (87.5%) focused on CPD as the prevailing continuing education model. One-third of the studies (33.3%) used the time-based model by accumulating continuing education hours, while almost half (41.7%) utilized the credit-based model by accumulating points. This review’s analysis of knowledge syntheses confirms that CPD encompasses various LLL activities, including formal and informal approaches. A quarter of the sampled articles (25%) used the number of entries per year classification. While blended learning has gained traction, a study found no significant difference in perceived and actual learning outcomes between online-only and blended approaches. Traditional face-to-face learning was highlighted as beneficial, either alone or in combination with blended or workplace learning. These findings contribute valuable insights into the evolving landscape of pharmacy CPD, shedding light on the varied approaches and their impact on learning outcomes.

Public health crises, such as pandemics or other health emergencies, can disrupt traditional modes of education delivery. Pharmacists may face challenges in attending in-person CPD events or adapting to sudden shifts in healthcare priorities [45]. Differences in the recognition of prior learning and work experience as valid components of CPD can be a challenge. Some countries may not fully acknowledge experiential learning, limiting the professional growth opportunities for seasoned pharmacists. Pharmacists in different countries may experience varying levels of workload and job-related stress. Balancing demanding work schedules with CPD requirements can contribute to professional burnout, impacting the enthusiasm and effectiveness of continued learning efforts [45]. 

In addressing these challenges, fostering collaboration among countries, sharing best practices, and developing internationally recognized standards for pharmacists’ CPD could contribute to a more cohesive and effective global approach to continuing professional development in pharmacy. These divergences carry profound implications for global continuing education effectiveness and inclusivity, warranting further research on LLL activities in pharmacy education. Collaborative efforts between educational institutions, professional organisations, and regulatory bodies may be crucial for aligning CPD goals with industry expectations.

This analysis of global continuing education and professional development for pharmacists reveals significant variations in LLL interventions and models. HICs and UMICs lead, while LMICs, LICs and some areas in the HICs face challenges. Variations impact healthcare services, professional mobility, and global inequalities. Tailored strategies considering economic contexts are essential. The study emphasises the multifaceted impact of variations on pharmacists’ professional development, highlighting potential compromises in patient care and challenges in workforce mobility. Understanding diverse models is crucial for inclusivity and effective global strategies. Blended learning, face-to-face, and workplace learning are all significant, emphasising the importance of diverse educational methods. Addressing challenges requires collaborative efforts, including knowledge exchange, capacity building, and internationally recognised standards. Collaboration between institutions, organisations, and regulatory bodies is crucial for aligning goals with industry expectations.

This scoping review highlights a scarcity of literature on preferences for models and effective LLL activities in CPE/CPD specific to pharmacists globally. Despite global recognition, limited works delineate patterns and preferences, emphasising the need for future research and collaborative initiatives to enhance pharmacist CPE/CPD universally. Recognising income disparities is crucial for formulating inclusive global strategies for pharmacist professional development. Tailoring initiatives to economic contexts can bridge gaps in education, ensuring equitable access. Collaborative efforts between high- and low- to middle-income countries can facilitate sustainable models, addressing unique challenges faced by pharmacists. The evolution of diverse CPE/CPD models globally highlights the leadership of high-income countries and emphasises the global trend toward mandatory CPD for pharmacists’ licensure. Ongoing research is essential to strengthen implementation, align the CPD model with evolving pharmacy profession needs, and support the progression of pharmacy practice in developing healthcare systems.

## Limitations

Several limitations should be acknowledged in this scoping review. Firstly, the absence of grey literature may be a limitation, and future studies may benefit from its inclusion (especially a review of regulator websites). To enhance organization, a cascading process could be employed to systematically build a structured list of key terms and sources for grey literature. Another limitation arises from the predominance of articles from HICs in the review due to language restrictions during electronic searches, particularly in English. This limitation may result in an incomplete representation of data from LMICs. Additionally, the temporal scope is limited to pre-COVID-19, potentially limiting the applicability of findings to current practices. While the global shift to online education is recognised, certain countries may still favor face-to-face delivery, as reflected in the referenced papers. In addition, it is too early to determine whether the COVID-19 practice changes will remain in effect post COVID-19.

Future research should prioritise exploring CPE/CPD in both LMICs and HICs, focusing on contexts with relevant CPD cycles, evaluations, reflections, and portfolio development. In-depth investigations into LLL activities, especially in rural areas and post-COVID-19 scenarios, are needed to enrich the existing evidence base. Furthermore, prospective research should explore online, blended, and emerging learning aspects, such as social networking and mobile applications, particularly in remote locations, to enhance learning outcomes [13, 22]. Workplace learning is also crucial for retention strategies among South African pharmacists [17]. 

## Conclusion

CPE/CPD are integral processes in the professional journey of pharmacists, emphasising ongoing education and skill enhancement for career advancement. These processes are vital for pharmacists to remain current with evolving practices, ensuring the maintenance of competency standards. The global shift towards CPD brings a positive influence by encouraging a reflective approach to learning needs and incorporating LLL interventions. This transition involves a blend of formal and informal methods for monitoring and documenting acquired skills, knowledge, and experiences. Despite the increasing integration of technology in education, face-to-face learning remains the preferred mode, with blended learning approaches following closely. However, structured planning for LLL activities is essential to meet mandatory statutory requirements and global pharmacist licensure standards. The diverse landscape of CPE/CPD models and regulatory prerequisites for pharmacist registration across nations underscores the necessity for a concerted effort towards global harmonisation. Such efforts aim to enhance clarity and ensure consistency in the professional development of pharmacists on an international scale.

## Data Availability

Not applicable.
